# Predictors of Maternal Vitamin D Deficiency and Associated Adverse Neonatal Outcomes in Eastern Uganda: A Cross‐Sectional Study

**DOI:** 10.1002/hsr2.72103

**Published:** 2026-03-15

**Authors:** Hussein Mire Hamdi, Marie Pascaline Sabine Ishimwe, Theodore Nteziyaremye, Kasujja Musa, Sowda Abdikarim Sheikh Isse, Geoffrey Okot, Emmanuel Okurut, Damulira Adam, Hassan Abdullahi Hafsa, Mohamud Mohamed Sadia, Ramla Abdi Ali, Adam Abdrahim Suliman Ebaid, Amos Muhumuza, Maxwell Okello, Ahmed Kiswezi Kazigo, Theoneste Hakizimana

**Affiliations:** ^1^ Department of Obstetrics and Gynecology Kampala International University Ishaka Uganda; ^2^ Department of Pediatrics and Child Health Kampala International University Ishaka Uganda; ^3^ Department of Sciences University of Rwanda Kigali Rwanda; ^4^ Department of Surgery Kampala International University Ishaka Uganda

**Keywords:** adverse neonatal outcomes, Eastern Uganda, maternal vitamin D deficiency, predictors

## Abstract

**Background and Aims:**

Vitamin D deficiency in pregnancy has been linked to adverse maternal and neonatal outcomes, but evidence from Uganda is limited. We estimated the prevalence of maternal vitamin D deficiency at delivery, identified associated factors, and assessed immediate neonatal outcomes.

**Methods:**

We conducted a hospital‐based cross‐sectional study among 315 postpartum women at Jinja Regional Referral Hospital (October 2024–January 2025). Serum 25‐hydroxyvitamin D was measured; deficiency was defined as < 20 ng/mL. Predictors were assessed using logistic regression. Neonatal outcomes were extracted from delivery records.

**Results:**

Vitamin D deficiency was present in 77.8% (245/315) of participants; mean 25(OH)D was 18.3 (SD 7.5) ng/mL. Urban residence, low educational attainment, higher BMI, fewer antenatal visits, and lack of regular exercise were independently associated with deficiency. Low 5‐min Apgar score was more frequent among neonates of deficient mothers.

**Conclusions:**

Maternal vitamin D deficiency was highly prevalent and associated with modifiable factors. Maternal deficiency was associated with poorer immediate neonatal indicators at birth; however, neonatal vitamin D levels were not measured and causality cannot be inferred.

**Abbreviations**
ANC: antenatal care
BMI: body mass index
IREC: Institutional Research & Ethics Committee.
JRRH: Jinja Regional Referral Hospital
NICU: neonatal intensive care unit
REC: Research Ethics Committee
SGA: small‐for‐gestational‐age
VD: vitamin D
VDD: vitamin D deficiency
VDR: vitamin D receptor
WHO: World Health Organization

## 1 Introduction

Vitamin D, historically recognized for its role in calcium‐phosphorus homeostasis and the prevention of rickets, has gained renewed attention for its broader physiological functions during pregnancy [1, 2]. Since its discovery in the early 20th century, vitamin D has been identified as a prohormone with receptors present in nearly all human tissues, influencing immune modulation, glucose metabolism, placental function, and fetal development [2, 3]. Despite abundant sunlight in many parts of sub‐Saharan Africa, vitamin D deficiency remains highly prevalent due to factors such as limited sun exposure, high melanin pigmentation, urbanization, poor dietary intake, and sociocultural practices [1, 4]. During pregnancy, maternal vitamin D requirements increase significantly to support fetal skeletal mineralization and immunological adaptation [5]. The placenta expresses vitamin D receptors and synthesizing enzymes, underscoring the crucial role of hormones in implantation and fetal growth [6, 7] Insufficient maternal vitamin D levels are associated with an increased risk of obstetric and neonatal complications, including preeclampsia, gestational diabetes mellitus, preterm birth, low birth weight (LBW), and neonatal asphyxia [8, 9].

Evidence from randomized trials also suggests that vitamin D supplementation during pregnancy may reduce the risk of pre‐eclampsia and other adverse outcomes, although effects can vary by baseline vitamin D status and setting [10]. However, despite these well‐documented associations, routine vitamin D screening and supplementation during pregnancy are not standard components of antenatal care in many low‐resource settings, including Uganda.

Globally, the prevalence of vitamin D deficiency among pregnant women varies widely, ranging from 18% to 84%, depending on geographic location, ethnicity, and diagnostic criteria [11, 12]. Studies have reported alarmingly high deficiency rates, with prevalence estimates of 81.7% in Ghana, over 90% in Malaysia, and up to 87% among laboring women in Iraq [13, 14, 15]. Similar findings have been documented in China, India, and Brazil [16, 17, 18] However, data from East Africa, particularly Uganda, remain scarce despite the region's high burden of maternal and neonatal morbidity.

In addition to its well‐established role in fetal skeletal development, vitamin D is also implicated in immunomodulation, uterine contractility, and placental vascularization, suggesting a potential influence on labor progression and birth outcomes [19, 20]

Several studies have linked maternal vitamin D deficiency to an increased risk of low Apgar scores, neonatal intensive care unit (NICU) admission, small‐for‐gestational‐age (SGA) births, and preterm delivery [21, 22].

Maternal and neonatal vitamin D concentrations are related, but neonatal vitamin D status cannot be assumed from maternal levels alone. In a prospective mother–infant cohort, maternal 25(OH)D correlated with neonatal concentrations, highlighting the need for direct neonatal measurement when neonatal vitamin D status is an outcome of interest [23].

Despite the growing evidence linking maternal vitamin D deficiency to adverse neonatal outcomes, Uganda lacks routine antenatal screening for vitamin D levels, and no national guidelines exist for supplementation. Additionally, populations with darker skin tones, such as those in Uganda, are biologically predisposed to lower endogenous vitamin D synthesis because of melanin's UV‐blocking effect [24]. Uganda continues to face a high neonatal mortality rate, estimated at 19 deaths per 1,000 live births [25] with prematurity, low birth weight, and asphyxia being major contributors. In the absence of local research, clinical guidelines remain generalized and may fail to address the specific needs of this high‐risk population.

This study aims to fill a critical knowledge gap by determining the prevalence, predictors, and neonatal consequences of maternal vitamin D deficiency in Eastern Uganda. The findings from this research will provide essential evidence to inform antenatal care policies and improve maternal and neonatal outcomes in the region.

## 2 Methods

### 2.1 Study Design and Setting

This hospital‐based cross‐sectional study was conducted in the postnatal ward of Jinja Regional Referral Hospital (JRRH) in eastern Uganda. Data collection took place over a 3‐month period, from October 2024 to January 2025. JRRH is a high‐volume tertiary healthcare facility that provides comprehensive emergency obstetric and neonatal care. It serves as a referral center for multiple districts and handles approximately 15–20 deliveries per day. The obstetrics and gynecology department comprises specialists, medical officers, postgraduate residents, nurses, and midwives. Additionally, the hospital is equipped with a modern laboratory capable of performing various diagnostic tests, including vitamin D tests.

### 2.2 Study Population

All women who delivered at Jinja Regional Referral Hospital (JRRH) and were admitted to the postnatal ward within 24 h of childbirth were eligible for inclusion, provided they gave written informed consent. We excluded women who were critically ill or unable to participate in an interview, those with multiple gestations (twins or triplets), and those with known chronic renal, liver, or parathyroid disease. For clarity, we also excluded women who reported current vitamin D supplementation or use of medications known to substantially affect vitamin D metabolism (e.g., long‐term anticonvulsants, rifampicin, or prolonged systemic glucocorticoids).

### 2.3 Sample Size Determination

The sample size was calculated using Kish‐Leslie formula (1965):

$$n=\frac{z^{2}p\left(1-p\right)}{e^{2}}$$

Using findings from a study in India where 75.3% of mothers had vitamin D deficiency [26], *p* = 0.753, e = 0.05 for a 95% level of confidence as well as Z = 1.96. On substituting, *n* = 286

When 10% was added to account for nonresponse and increase internal validity, the sample size required was 315.

Therefore, the minimum sample size required for this study was 315 women in immediate postpartum period.

### 2.4 Sampling Technique

A systematic random sampling method was used to select participants. Hospital records indicated that approximately 1,500 women were admitted to the postnatal ward during the 3‐month study period. To achieve the target sample size of 315, a sampling interval of four was determined. Accordingly, every fourth eligible postpartum woman was approached for enrollment. If a selected participant declined to participate, the next eligible woman was recruited, after which the systematic interval resumed. This approach ensured consistent sampling and minimized selection bias.

### 2.5 Data Collection Procedure

All women admitted to the postnatal ward of JRRH within 24 h of childbirth were informed about the study and provided written informed consent. Data were collected via a structured, interviewer‐administered questionnaire that was available in both English and the local language. Blood samples were taken for vitamin D testing, and adverse neonatal outcomes were extracted from delivery and neonatal records.

### 2.6 Study Variables

The independent variables were sociodemographic, clinical, and obstetric factors associated with vitamin D deficiency. The dependent variable was vitamin D deficiency, which was tested in the laboratory. Adverse neonatal outcomes reflected as secondary outcomes included stillbirth, small for gestational age, preterm birth and the APGAR score.

### 2.7 Vitamin D Testing

A 4 mL venous blood sample was collected from the antecubital fossa using a serum separator tube (Becton Dickinson, USA). The samples were stored at low temperature and later analysed using the Cobas E601 electrochemiluminescence binding assay (Roche Diagnostics), following standardized laboratory procedures. Serum 25‐hydroxyvitamin D [25(OH)D] concentrations were recorded in ng/mL. Deficiency was defined as < 20 ng/mL.

### 2.8 Quality Control

The data collection tools were pretested in both English and the local language to ensure clarity and cultural appropriateness. Research assistants underwent rigorous training and were supervised throughout the study by the principal investigator and a supervising gynecologist to ensure consistency and adherence to ethical and procedural standards. During data collection, completed questionnaires were routinely cross‐checked for completeness and accuracy. For laboratory analysis, all vitamin D assays were performed under strict quality control protocols, including routine instrument calibration and control runs prior to sample testing. Additionally, every 20th sample was tested independently at Kampala International University Teaching Hospital to assess the reliability of the results. This cross‐sectional observational study is reported in accordance with STROBE reporting principles.

### 2.9 Data Management and Analysis

Data were entered into Microsoft Excel and exported to Stata version 15 (StataCorp, College Station, TX, USA) for analysis. Continuous variables (including serum 25‐hydroxyvitamin D) were summarized using means with standard deviations or medians with interquartile ranges, as appropriate, while categorical variables were summarized using frequencies and percentages. The prevalence of vitamin D deficiency was estimated with 95% confidence intervals (CIs).

The primary analysis evaluated factors associated with maternal vitamin D deficiency. Univariable logistic regression was used to estimate crude odds ratios (cORs) with 95% CIs. Variables with *p* ≤ 0.20 in Univariable analysis and those considered clinically relevant were entered into a multivariable logistic regression model to estimate adjusted odds ratios (aORs) with 95% CIs.

Neonatal outcomes were analysed as secondary outcomes. Neonatal outcomes were compared by maternal vitamin D status using chi‐square or Fisher's exact tests, and logistic regression was used to estimate odds ratios with 95% CIs for selected outcomes. All statistical tests were two‐sided with an a priori significance level of α = 0.05.

### 2.10 Human Ethics and Consent to Participate

This study received ethical clearance from the Kampala International University Research Ethics Committee (KIU‐REC), under registration number KIU‐2024‐445. Written informed consent was obtained from all participants prior to enrolment. The research was conducted in accordance with the ethical principles outlined in the Declaration of Helsinki. Confidentiality and anonymity were strictly upheld throughout the study.

## 3 Results

### 3.1 Basic Characteristics of the Study Participants

This was a hospital‐based cross‐sectional study that recruited a total of 315 postnatal mothers. The majority of the participants were aged between 20 and 30 years 180 (57.14%), 158 (50.16%) were from urban areas and 258 (81.90%) were married. Most of the participants had attained at least a secondary education level 111 (35.24%), didn't smoke 293 (93.02%) and earned less than 200,000UGX 147 (46.67%) (Table 1).

**Table 1:** Sociodemographic characteristics of the participants (*N* = 315).

Variables	Frequency (*n*)	Percentage (%)
**Age**		
< 20 years	78	24.76
20–30 years	180	57.14
≥ 30 years	57	18.10
**Residence**		
Rural	157	49.84
Urban	158	50.16
**Marital status**		
Not married	57	18.10
Married	258	81.90
**Education level**		
None	92	29.21
Primary	84	26.67
Secondary	111	35.24
Tertiary	28	8.89
**Smoking**		
Yes	22	6.98
No	293	93.02
**Income level (UGX)**		
< 200,000	147	46.67
200,000–500,000	134	42.54
> 500,000	34	10.79

### 3.2 Prevalence of Vitamin D Deficiency Among Mothers Delivered in Eastern Uganda

**Figure 1:**
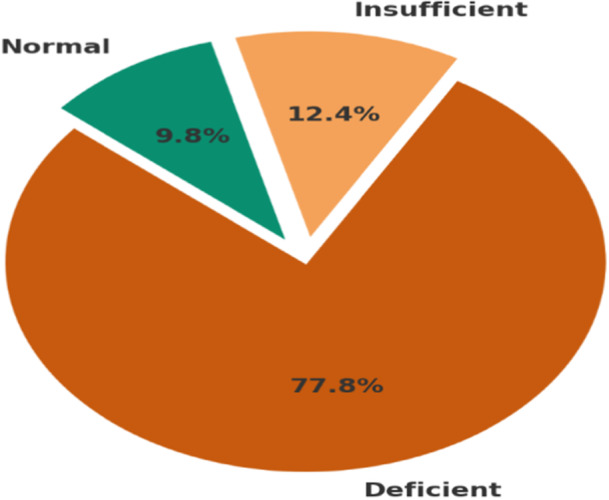
A pie chart illustrating the proportion of participants with vitamin D deficiency (*N* = 315).

Among the 315 postpartum women enrolled in the study, 77.8% (*n* = 245; 95% CI: 72.8%–82.1%) were classified as vitamin D deficient (serum 25[OH]D < 20 ng/mL), whereas 12.4% (*n* = 39) had insufficient levels (20.0–29.9 ng/mL), and only 9.8% (*n* = 31) had sufficient vitamin D status (≥ 30 ng/mL). The overall mean serum 25(OH)D concentration was 18.3 ng/mL (SD = 7.5), with a median of 18.0 ng/mL and an interquartile range (IQR) of 12.2–22.8 ng/mL (Figure 1).

### 3.3 Factors Associated With Vitamin D Deficiency Among Women Delivered in Eastern Uganda

We employed both bivariable and multivariable analysis where factors with *p* values less than 0.2 in the bivariable analysis including residence, marital status, education level, income level, BMI and exercise were considered in the multivariable analysis. The multivariable analysis revealed that women living in urban areas were 4 times more likely to develop vitamin D deficiency. Women with no formal education were approximately five times more likely to have Vitamin D deficiency, whereas those with only primary education were nearly four times more likely to have vitamin D deficiency than those with tertiary education.

Body mass index showed a clear pattern, overweight women were more than five times more likely to have vitamin D deficiency, and obese women were nearly seven times more likely to have vitamin D deficiency than women with a normal BMI. Antenatal care attendance was strongly associated with vitamin D status; women who attended fewer than four visits had approximately seven times the odds of vitamin D deficiency, and those with 4–7 visits had over three times the odds compared with women who attended eight or more visits

A lack of regular exercise was linked to a sixfold increase in the likelihood of vitamin D deficiency. Other factors, including age, marital status, income, smoking status, and dietary balance, were not significantly associated with vitamin D deficiency. (Table 2).

**Table 2:** Factors Associated with Vitamin D deficiency among women delivered in Eastern Uganda (*N* = 315).

Variables	No deficiency (*n*, %)	Deficient (*n*, %)	COR (95% CI)	*p* value (COR)	AOR (95% CI)	*p* value (AOR)
**Age**						
< 20 years	18 (25.7)	60 (24.5)	1.00		1.00	
20–34 years	39 (55.7)	141 (57.6)	1.08 (0.57–2.05)	0.802	2.25 (0.97–5.22)	0.058
≥ 35 years	13 (18.6)	44 (18.0)	1.02 (0.45–2.29)	0.971	2.39 (0.80–7.11)	0.117
**Residence**						
Rural	46 (65.7)	111 (45.3)	1.00		1.00	
Urban	24 (34.3)	134 (54.7)	2.31 (1.33–4.03)	0.003	4.00 (1.84–8.70)	**< 0.001**
**Marital status**						
Married	53 (75.7)	205 (83.7)	1.00		1.00	
Not married	17 (24.3)	40 (16.3)	0.61 (0.32–1.16)	0.130	0.55 (0.24–1.23)	0.145
**Education level**						
None	11 (15.7)	81 (33.1)	6.38 (2.41–16.89)	< 0.001	5.05 (1.59–15.99)	**0.006**
Primary	17 (24.3)	67 (27.3)	3.42 (1.37–8.52)	0.008	3.73 (1.22–11.42)	**0.021**
Secondary	29 (41.4)	82 (33.5)	2.45 (1.04–5.76)	0.040	2.34 (0.74–7.35)	0.147
Tertiary	13 (18.6)	15 (6.1)	1.00		1.00	
**Smoking**						
No	68 (97.1)	225 (91.8)	1.00		1.00	
Yes	2 (2.9)	20 (8.2)	3.02 (0.69–13.26)	0.143	1.27 (0.24–6.65)	0.774
**Income level (,000 UGX)**						
< 200	27 (38.6)	120 (49.0)	2.12 (0.93–4.88)	0.075	2.22 (0.75–6.59)	0.150
200–500	32 (45.7)	102 (41.6)	1.52 (0.67–3.46)	0.314	0.92 (0.26–3.19)	0.896
> 500	11 (15.7)	23 (9.4)	1.00		1.00	
**BMI**						
Underweight	5 (7.1)	34 (13.9)	2.73 (1.01–7.36)	0.047	2.48 (0.61–9.97)	0.202
Normal	53 (75.7)	132 (53.9)	1.00		1.00	
Overweight	8 (11.4)	34 (13.9)	1.71 (0.74–3.93)	0.209	5.43 (1.77–16.68)	**0.003**
Obese	4 (5.7)	45 (18.4)	4.51 (1.55–13.18)	0.002	6.71 (1.99–22.64)	**0.002**
**Antenatal visits**						
< 4 visits	21 (30.0)	110 (44.9)	2.97 (1.46–6.05)	0.003	6.99 (2.19–22.33)	**0.001**
4–7 visits	28 (40.0)	98 (40.0)	1.99 (1.01–3.92)	0.048	3.19 (1.19–8.61)	**0.022**
≥ 8 visits	21 (30.0)	37 (15.1)	1.00 (Ref)		1.00	
**Balanced diet**						
Yes	31 (44.3)	81 (33.1)	1.00		1.00	
No	39 (55.7)	164 (66.9)	1.61 (0.94–2.77)	0.085	1.35 (0.68–2.68)	0.398
**Exercise**						
Yes	54 (77.1)	144 (58.8)	1.00		1.00	
No	16 (22.9)	101 (41.2)	2.37 (1.28–4.37)	0.006	6.06 (2.37–15.46)	**< 0.001**

Abbreviations: aOR, adjusted odds ratio; cOR, crude odds ratio.

### 3.4 Adverse Neonatal Outcomes of Mothers With Vitamin D Deficiency

Compared with mothers with sufficient vitamin D, neonates born to vitamin D–deficient mothers had a significantly higher incidence of low Apgar scores at 5 min (17.6% vs. 7.1%, *OR* = 2.77, *p* = 0.033). Preterm birth was also more common among the deficient group (20.0% vs. 10.0%, *OR* = 2.25), with the difference approaching statistical significance (*p *= 0.054). Although not statistically significant, small for gestational age and stillbirth were more common in the deficient group. Stillbirth and small‐for‐gestational‐age births were uncommon but occurred more often among deficient mothers (Table 3).

**Table 3:** Adverse Neonatal Outcomes by Maternal Vitamin D Status (*N* = 315). (After adjustment for patients characteristics in Table 2).

Outcome	Deficient (*N* = 245)	Non–deficient (*N* = 70)	aOR (95%CI)	*p* value
SGA	17 (6.9%)	2 (2.9%)	2.54 (0.57–11.25)	0.206
Stillbirth	13 (5.3%)	1 (1.4%)	3.87 (0.50–30.08)	0.16
Low Apgar score at 5 min	43 (17.6%)	5 (7.1%)	2.77 (1.05–7.28)	0.033*
Preterm birth	49 (20.0%)	7 (10.0%)	2.25 (0.97–5.22)	0.054

* Statistically significant, small for gestational age (SGA).

## 4 Discussion

This study revealed a strikingly high prevalence of vitamin D deficiency (77.8%) among postpartum women in Uganda. This rate aligns with findings from similar low‐resource settings where the prevalence ranges from 51.3% to 100% [11]. Studies from India report comparably high rates of 75.3% [26] and 70.7% [12], highlighting that in many low‐ and middle‐income countries, deficiency is a pervasive issue. The consistency of these findings is likely due to shared factors such as low dietary intake, limited sun exposure due to indoor occupations or cultural practices, lack of fortified foods, and economic barriers to supplementation.

Comparable data from China also support these results. For example, Chen et al., reported that 83.3% of pregnant women had deficient vitamin D levels [16], whereas Hong‐Bi et al., reported nearly 90% deficiency [22]. These populations, like Uganda's population, are affected by urbanization, traditional clothing, and limited dietary diversity. Conversely, studies from high‐income countries reveal lower deficiency rates, such as 13% in Denmark [21] and 19.1% in Brazil [18], largely due to systematic supplementation, antenatal nutrition counselling, and food fortification programs.

Our findings also parallel those from Iraq [15] and Iran where vitamin D deficiency affects more than 80% of participants [27]. These consistent patterns underscore the global health challenge posed by vitamin D insufficiency, particularly in low‐resource settings with limited health infrastructure and public health interventions.

Several key factors independently predict vitamin D deficiency. Women residing in urban areas were four times more likely to have vitamin D deficiency than their rural counterparts were. This association may be explained by reduced sun exposure due to indoor lifestyles, high‐rise living, and air pollution, as noted in India and Malaysia [7, 28]. Similar findings were reported in Brazil [17], highlighting that urbanization, despite access to healthcare, may contribute to suboptimal vitamin D synthesis.

Lower educational attainment significantly increased the risk of deficiency, with women lacking formal education having over five times higher odds than those with tertiary education. Limited awareness of nutritional needs, poor access to health information, and inadequate dietary practices may contribute to this trend, which has also been observed in studies from Belgium and Iraq [15, 29]

Increased body mass index was a strong predictor. Compared with women with normal BMI, obese women had nearly seven times higher odds of deficiency compared to those with normal BMI. This finding is consistent with findings from India and China, where adiposity reduces circulating vitamin D due to its sequestration in adipose tissue [16, 26].

Inadequate antenatal care has emerged as a critical factor. Women with fewer than four antenatal visits had seven times higher odds of deficiency. This likely reflects missed opportunities for screening, supplementation, and health education [17]. This finding also underscores the importance of integrating vitamin D supplementation into routine antenatal care.

Finally, physical inactivity was associated with a sixfold increased risk of deficiency. Limited outdoor activity reduces UVB‐mediated synthesis of vitamin D, a relationship supported by Woon et al., in Malaysia [28] and Aggarwal et al., in India [7].

These findings indicate that vitamin D deficiency is driven by a combination of socioeconomic, behavioral, and environmental factors. Addressing these determinants through education, antenatal care strengthening, and lifestyle interventions is essential.

This study revealed that maternal vitamin D deficiency was significantly associated with adverse neonatal outcomes. Notably, low Apgar scores at 5 min were more common among infants of deficient mothers (17.6% vs. 7.1%, *p* = 0.033), as well as preterm birth (20.0% vs. 10.0%, *p* = 0.054) though not statistically significant. Although stillbirth and small‐for‐gestational‐age (SGA) differences were not statistically significant, the observed trends are clinically important.

These results align with global evidence linking maternal Hypovitaminosis D to poor neonatal outcomes. For example, Bergløv et al., reported increased risks of preterm birth and SGA in vitamin D–deficient HIV‐positive women [21], whereas Woo et al., reported a 3.3‐fold increased risk of preterm birth among deficient African‐American women [24]. Studies in China and the Netherlands also linked low maternal vitamin D to fetal growth restriction, reduced head circumference, and low birth weight [16, 22, 30].

Other studies, however, reported inconsistent associations. For example, Nageshu et al., and Sooriyaarachchi et al., did not observe significant differences in neonatal outcomes according to maternal vitamin D status [31, 32]. Such variability could be due to differences in study design, population characteristics, cut‐off points for deficiency, or sample sizes.

Our findings show an association between maternal vitamin D deficiency and selected immediate neonatal outcomes recorded at delivery. However, we did not measure neonatal 25(OH) D concentrations; therefore, neonatal vitamin D status and mechanisms cannot be determined from this study. The observed associations should be interpreted cautiously given the cross‐sectional design and potential residual confounding.

### 4.1 Strengths and Limitations of the Study

To the best of our knowledge, this is the first study in eastern Uganda to quantify maternal vitamin D deficiency at delivery and examine its association with immediate neonatal outcomes, providing baseline data for future research and policy. Key strengths include biochemical measurement of maternal 25(OH)D and systematic collection of maternal and neonatal clinical data.

However, the cross‐sectional design limits inference on temporality and causality between maternal vitamin D status and neonatal outcomes. Some relevant exposures were not fully measured, including detailed dietary intake, objective measures of sun exposure, and comprehensive supplementation/medication history (including short‐course antibiotics), which may result in residual confounding. In addition, neonatal 25(OH)D concentrations were not measured, and neonatal outcomes were derived from routine delivery records, which may be subject to incomplete documentation.

## 5 Conclusions

Vitamin D deficiency was highly prevalent among women delivering at Jinja Regional Referral Hospital. Urban residence, lower education, higher BMI, fewer antenatal visits, and lack of exercise were associated with deficiency. Maternal deficiency was associated with poorer immediate neonatal outcomes at birth (notably low 5‐min Apgar scores), but neonatal vitamin D levels were not measured and causality cannot be inferred. Prospective studies measuring mother–infant vitamin D and supplementation exposure are needed to guide policy on screening and supplementation in Uganda. These observations emphasize the clinical importance of optimizing maternal vitamin D status through targeted interventions such as routine screening and appropriate supplementation to improve neonatal outcomes. We recommend that the Ministry of Health revise antenatal care protocols to include systematic vitamin D screening, ensuring early detection and effective management of deficiency. Public health strategies should target modifiable determinants by providing comprehensive nutritional education, advocating for safe sun exposure practices, promoting regular physical activity, and increasing antenatal care participation. Furthermore, the establishment of standardized vitamin D supplementation guidelines within maternal health programs is recommended, alongside the implementation of longitudinal studies to assess the extended benefits of improved maternal vitamin D status on neonatal outcomes.

## Author Contributions

Conceptualization: Hussein Mire Hamdi and Theoneste Hakizimana. Data analysis: Theoneste Hakizimana, Marie Pascaline Sabine Ishimwe and Theodore Nteziyaremye. Supervision: Theoneste Hakizimana, Ahmed Kiswezi Kazigo and Damulira Adam. Visualization: Hussein Mire Hamdi, Theodore Nteziyaremye and Maxwell Okello. Writing – original draft: Hussein Mire Hamdi, Marie Pascaline Sabine Ishimwe and Sowda Abdikarim Sheikh Isse. Writing–review and editing: Kasujja Musa, Geoffrey Okot, Emmanuel Okurut, Hassan Abdullahi Hafsa, Mohamud Mohamed Sadia, Ramla Abdi Ali, Adam Abdrahim Suliman Ebaid, Amos Muhumuza, Ahmed Kiswezi Kazigo, Theodore Nteziyaremye and Theoneste Hakizimana. All authors read and approved the final manuscript.

## Funding

This research received no specific grant from any funding agency in the public, commercial, or not‐for‐profit sectors. No funding body had any role in study design; data collection, analysis, or interpretation; writing of the report; or the decision to submit for publication.

## Consent

The authors have nothing to report.

## Conflicts of Interest

The authors declare no conflicts of interest.

## Transparency Statement

The lead author Theoneste Hakizimana affirms that this manuscript is an honest, accurate, and transparent account of the study being reported; that no important aspects of the study have been omitted; and that any discrepancies from the study as planned (and, if relevant, registered) have been explained.

## Data Availability

The data that support the findings of this study are available from the corresponding author Dr. Theoneste Hakizimana (email: theonestehakizimana5@gmail.com) upon reasonable request. De‐identified data can be shared in line with institutional ethics approvals and participant confidentiality protections.

## References

[1] A. Augoulea , G. Zachou , C. Giakoumi , M. Moros , K. Panoulis , and I. Lambrinoudaki , “Maternal Vitamin D Deficiency and Adverse Pregnancy Outcomes,” Gynecological and Reproductive Endocrinology & Metabolism 1 (2020): 95–103, 10.53260/grem.201025.

[2] M. F. Holick , “The One‐Hundred‐Year Anniversary of the Discovery of the Sunshine Vitamin D3: Historical, Personal Experience and Evidence‐Based Perspectives,” Nutrients 15 (2023): 593. 36771300 10.3390/nu15030593 PMC9919777

[3] H. Zhang , S. Wang , L. Tuo , Q. Zhai , and D. Xu , “Relationship Between Maternal Vitamin D Levels and Adverse Outcomes,” Nutrients 14 (2022): 1–18. 10.3390/nu14204230 PMC9610169 36296914

[4] A. Arabi , R. El Rassi , and G. El‐Hajj Fuleihan , “Hypovitaminosis D in Developing Countries—Prevalence, Risk Factors and Outcomes,” Nature Reviews Endocrinology 6 (2010): 550–561, 10.1038/nrendo.2010.146. 20852586

[5] E. M. Curtis , R. J. Moon , N. C. Harvey , and C. Cooper , “Maternal Vitamin D Supplementation During Pregnancy,” British Medical Bulletin 126 (2018): 57–77, 10.1093/bmb/ldy010.Maternal. 29684104 PMC6003599

[6] C. L. Wagner and B. W. Hollis , “The Implications of Vitamin D Status During Pregnancy on Mother and Her Developing Child,” Frontiers in Endocrinology 9 (2018): 500, 10.3389/fendo.2018.00500. 30233496 PMC6127214

[7] N. Aggarwal , R. Singla , U. Dutta , et al., “Prevalence of Vitamin D Deficiency Among Pregnant Women and Effect of Vitamin D Supplementation on Maternal and Fetal Outcomes: A Double‐Blind Randomized Placebo Controlled Trial,” Asian Journal of Medical Sciences 13 (2022): 95–101, 10.3126/ajms.v13i2.40639.

[8] M. F. C. Akdulum and K. Ö. Biberoğlu , “Does Maternal Vitamin D Deficiency Affect Perinatal Outcomes?,” Chronicles of Precision Medical Researchers 4 (2023): 45–49, 10.5281/zenodo.7715692.

[9] M. Chakhtoura , A. Nassar , A. Arabi , et al., “Effect of Vitamin D Replacement on Maternal and Neonatal Outcomes: A Randomised Controlled Trial in Pregnant Women With Hypovitaminosis D. A Protocol,” BMJ Open 6 (2016): e010818, 10.1136/bmjopen-2015-010818. PMC4785305 26956166

[10] A. Kokkinari , E. Antoniou , E. Orovou , et al., “The Role of Vitamin D Supplementation in Preventing Pre‐Eclampsia: A Review of Randomized Controlled Trials With Meta‐Analysis,” Healthcare 13 (2025): 1221, 10.3390/healthcare13111221. 40508834 PMC12154485

[11] P. Pligt , Van Der , J. Willcox , and E. A. Szymlek‐gay , “Associations of Maternal Vitamin D Deficiency With Pregnancy and Neonatal Complications in Developing Countries: A Systematic Review,” Nutrients 14 (2018): 1–22. 10.3390/nu10050640 PMC5986519 29783717

[12] P. Kumar , A. Shenoi , R. K. Kumar , S. V. Girish , and S. Subbaiah , “Vitamin D Deficiency Among Women in Labor and Cord Blood of Newborns,” Indian Pediatrics 52 (2015): 530–531, 10.1139/apnm-2013-0579. 26121736

[13] L. A. Fondjo , W. Tashie , W. K. B. A. Owiredu , E. A. Adu‐gyamfi , and L. Seidu , “High Prevalence of Vitamin D Deficiency Among Normotensive and Hypertensive Pregnant Women in Ghana,” BMC Pregnancy and Childbirth 21 (2021): 331. 33902494 10.1186/s12884-021-03802-9 PMC8077698

[14] S. M. Saffian , N. A. Jamil , and E. Hatah , “Vitamin D Insufficiency is High in Malaysia: A Systematic Review and Meta‐Analysis of Studies on Vitamin D Status in Malaysia,” Frontiers in Nutrition 9 (2022): 1–13, 10.3389/fnut.2022.1050745. PMC9715981 36466384

[15] W. Faik , A. Mahfooth , R. O. Lafta , and A. N. Khuoo , “A Study of Vitamin D Level in Pregnancy and the Effect of Its Deficiency on Pregnancy Outcome,” Journal of Women's Health Care 9 (2020): 1–6, 10.35248/2167-0420.20.9.500.

[16] B. Chen , Y. Chen , and Y. Xu , “Vitamin D Deficiency in Pregnant Women,” Medicine (Baltimore) 100, no. 41 (2021): 1–7. 10.1097/MD.0000000000027505 PMC8519205 34731133

[17] A. Dave , M. Verma , N. Jain , and A. Dave , “A Study of Vitamin D Levels and Associated Deficiency in Pregnancy and Its Effect on Maternal and Fetal Outcome,” International Journal of Reproduction, Contraception, Obstetrics and Gynecology 6 (2016): 84–88.

[18] M. Martins , C. Esmeraldo , J. Sabiá , J. Carvalho , F. I. Suano‐Souza , and R. O. S. Sarni , “Vitamin D Postpartum Concentrations: Relationship With Nutritional Condition and Morbidities During Pregnancy,” Journal of Pregnancy 2018 (2018): 1070528, 10.1155/2018/1070528. 30245881 PMC6139194

[19] E. Larqué , E. Morales , R. Leis , and J. E. Blanco‐Carnero , “Maternal and Foetal Health Implications of Vitamin D Status During Pregnancy,” Annals of Nutrition and Metabolism 72 (2018): 179–192, 10.1159/000487370. 29533937

[20] L. Tzu‑Hui , T. Wu , P. Lib , and D. Ding , “Effect of Vitamin D Supplementation During Pregnancy on Maternal and Perinatal Outcomes,” Medical Journal 31 (2019): 201–206, 10.4103/tcmj.tcmj. PMC6905233 31867246

[21] A. Bergløv , E. Moseholm , T. L. Katzenstein , et al., “Prevalence and Association With Birth Outcomes of Low Vitamin D Levels Among Pregnant Women Living With HIV,” AIDS 35 (2021): 1491–1496, 10.1097/QAD.0000000000002899. 33813556

[22] S. Hong‐bi , X. Yin , Y. Xiaowu , et al., “High Prevalence of Vitamin D Deficiency in Pregnant Women and Its Relationship With Adverse Pregnancy Outcomes in Guizhou, China,” Journal of International Medical Research 46 (2018): 4500–4505, 10.1177/0300060518781477. 30270806 PMC6259374

[23] M. Jerković Raguž , T. Barišić , I. Mikulić , V. Mikulic , I. Šušak , and V. Tomic , “The Correlation of Vitamin D Concentrations in Healthy Pregnant Women and Their Infants With Outcome Parameters,” Zeitschrift für Geburtshilfe und Neonatologie 229 (2025): 274–279, 10.1055/a-2542-2818. 40068910

[24] J. Woo , T. Guffey , R. Dailey , D. Misra , and C. Giurgescu , “Vitamin D Status as an Important Predictor of Preterm Birth in a Cohort of Black Women,” Nutrients 15 (2023): 4637. 37960290 10.3390/nu15214637 PMC10649077

[25] Uganda Bureau of Statistics. Statistical Abstract. 2022.

[26] M. Menon , S. T. A., T. Mohan , and A. B. Patil , “Vitamin D Deficiency and Its Correlation With Pregnancy Outcome,” International Journal of Reproduction, Contraception, Obstetrics and Gynecology 9 (2020): 1493–1497.

[27] M. Ghafarzadeh , A. Shakarami , F. Tarhani , and F. Yari , “Evaluation of the Prevalence of Vitamin D DeFiciency in Pregnant Women and Its Correlation With Neonatal Vitamin D Levels,” Clinical Nutrition Open Science 36 (2021): 91–97, 10.1016/j.nutos.2021.02.007.

[28] F. C. Woon , Y. S. Chin , I. H. Ismail , et al., “Vitamin D Deficiency During Pregnancy and Its Associated Factors Among Third Trimester Malaysian Pregnant Women,” PLOSONE 14 (2019): e0216439. 10.1371/journal.pone.0216439 PMC6590777 31233513

[29] S. Vandevijvere , S. Amsalkhir , H. Van Oyen , and R. Moreno‐Reyes , “High Prevalence of Vitamin D Deficiency in Pregnant Women: A National Cross‐Sectional Survey,” PLoS One 7 (2012): e43868, 10.1371/journal.pone.0043868. 22937114 PMC3427250

[30] K. Miliku , A. Vinkhuyzen , L. M. Blanken , et al., “Maternal Vitamin D Concentrations During Pregnancy, Fetal Growth Patterns and Risks of Adverse Birth Outcomes,” American Journal of Clinical Nutrition 103 (2016): 1514–1522, 10.3945/ajcn.115.123752.Maternal. 27099250 PMC5410992

[31] S. Nageshu , K. Krishna , K. L., B. Bhat , H. Suma , and S. Reddy , “A Study of Prevalence of Vitamin D Deficiency Among Pregnant Women and Its Impact on Feto Maternal Outcome,” International Journal of Reproduction, Contraception, Obstetrics and Gynecology 5 (2016): 1174–1180.

[32] P. Sooriyaarachchi , D. T. Jeyakumar , N. King , and R. Jayawardena , “Clinical Nutrition ESPEN Impact of Vitamin D Deficiency on COVID‐19,” Clinical Nutrition ESPEN 44 (2021): 372–378, 10.1016/j.clnesp.2021.05.011. 34330492 PMC8164501

